# Rapid fabrication of sieved microwells and cross-flow microparticle trapping

**DOI:** 10.1038/s41598-020-72700-5

**Published:** 2020-09-24

**Authors:** Lauren Romita, Shyan Thompson, Dae Kun Hwang

**Affiliations:** 1 Department of Chemical Engineering, Ryerson University, 350 Victoria Street, Toronto, ON M5B 2K3 Canada; 2 Keenan Research Centre for Biomedical Science, St. Michael’s Hospital, 30 Bond Street, Toronto, ON M5B 1W8 Canada; 3 Institute for Biomedical Engineering, Science and Technology (iBEST), A Partnership Between Ryerson University and St. Michael’s Hospital, 30 Bond Street, Toronto, ON M5B 1W8 Canada

**Keywords:** Engineering, Materials science, Biomaterials, Materials for devices, Techniques and instrumentation

## Abstract

The use of microwells is popular for a wide range of applications due to its’ simplicity. However, the seeding of conventional microwells, which are closed at the bottom, is restricted to gravitational sedimentation for cell or particle deposition and therefore require lengthy settling times to maximize well occupancy. The addition of microfluidics to the capture process has accelerated cell or particle dispersion and improved capture ability but is mostly limited to gravitationally-driven settling for capture into the wells. An alternative approach to conventional closed-microwells, sieved microwells supersedes reliance on gravity by using hydrodynamic forces through the open pores at the bottom of the microwells to draw targets into the wells. We have developed a rapid fabrication method, based on flow lithography techniques, which allows us to easily customize the mesh pore sizes in a simple two-step process. Finally, by combining this microwell design with cross-flow trapping in a microfluidic two-layered channel, we achieve an 88 ± 6% well occupancy in under 10 s.

## Introduction

Microwells have been utilized for numerous biological and non-biological applications such as single cell analysis^1–6^, cell aggregation^7–13^ and anti-counterfeiting applications^14,15^. In particular, microwells have become a popular platform used to separate single cells or particles because of their simplicity, high sensitivity and ease of analysis^16–19^. For cellular experiments, each cell can be studied individually providing insights into the heterogeneity of the cell population^20–23^.

Although microwells have a simple design, the size and shape of the wells becomes an important factor for the effective trapping of single cells or particles^24,25^. The shape and diameter of the wells usually complements the characteristic features of the cells or particles, and the depth of the well is carefully selected to control the number of cells or particles that can settle into the well. The well must be deep enough to prevent washout but not too deep as to avoid multiple cells or particles from stacking in the well.

Microwells are typically seeded by pipetting a small sample of cells or particles over the surface of the wells and allowing them to settle over a period of time^24^. This step is normally repeated several times to maximize occupancy, resulting in a time-consuming process. In this pipet-seeding, gravity is the main driving force for sedimentation to trap targets, while the depth of the well controls the settling time required^26^. Instead of microwells with closed bottoms, adapting porous microwells in combination with negative pressure can enhance this sedimentation and trapping^15^, although the fabrication of porous membranes is a lengthy process and challenging.

Microfluidics have been integrated with microwells to expedite sample seeding and sedimentation, and improve capture efficiency because microfluidics offers high throughput and small sample size^27–30^. Recently, in a PDMS channel configured with spherical domes fabricated by liquid metal droplets, a hydrodynamic manipulation of cells inside the domes has been demonstrated^31^. Using a microfluidic channel over the microwells reduces the sedimentation time since the cells or particles are more evenly dispersed over the surface of the wells, in addition to being nearer to the wells because of the low channel height^32,33^. Regardless, time is still required for the cells or particles to fully sediment into the wells since gravity is the main force driving them to the bottom of said wells^34^. Optimizing flow pattern inside wells improves cell trapping; but intricate considerations for optimal well design and studies are unavoidable^26,35^. Alternatively, using porous microwells opposed to closed ones, in combination with a cross-flow configuration, can take full advantage of microwell-integrated microfluidic platforms since flow-guided targets can be directly trapped into the wells. However, besides the difficulty to fabricate such porous microwells that have ability to trap targets, it is challenging to assemble them into a microfluidic device enabling cross-flow.

Here we develop a rapid and high-throughput fabrication method for sieved microwells and introduce a successful route to integrate the sieved microwells into a double-layered microfluidic device to enable cross-flow trapping. We make microwells consisting of two layers: a bottom mesh layer and a top well layer, via two continuous steps based on slit-channel lithography in a microfluidic PDMS step channel. We install resulting sieved microwells into the conjunction site of a double-layered PDMS channel by flowing or sandwiching the microwells, allowing for direct-flow guided trapping. As a proof of concept demonstration, we test trapping performance of both microwell devices with disk-shaped particles. The permeable wells act as a sieve, allowing fluid to pass through the open-bottom of the wells while preventing the particles from following. This design eliminates the need for a sedimentation time since the hydrodynamic flow through the wells is the main driving force for particle capture.

## Results and discussion

**Figure 1:**
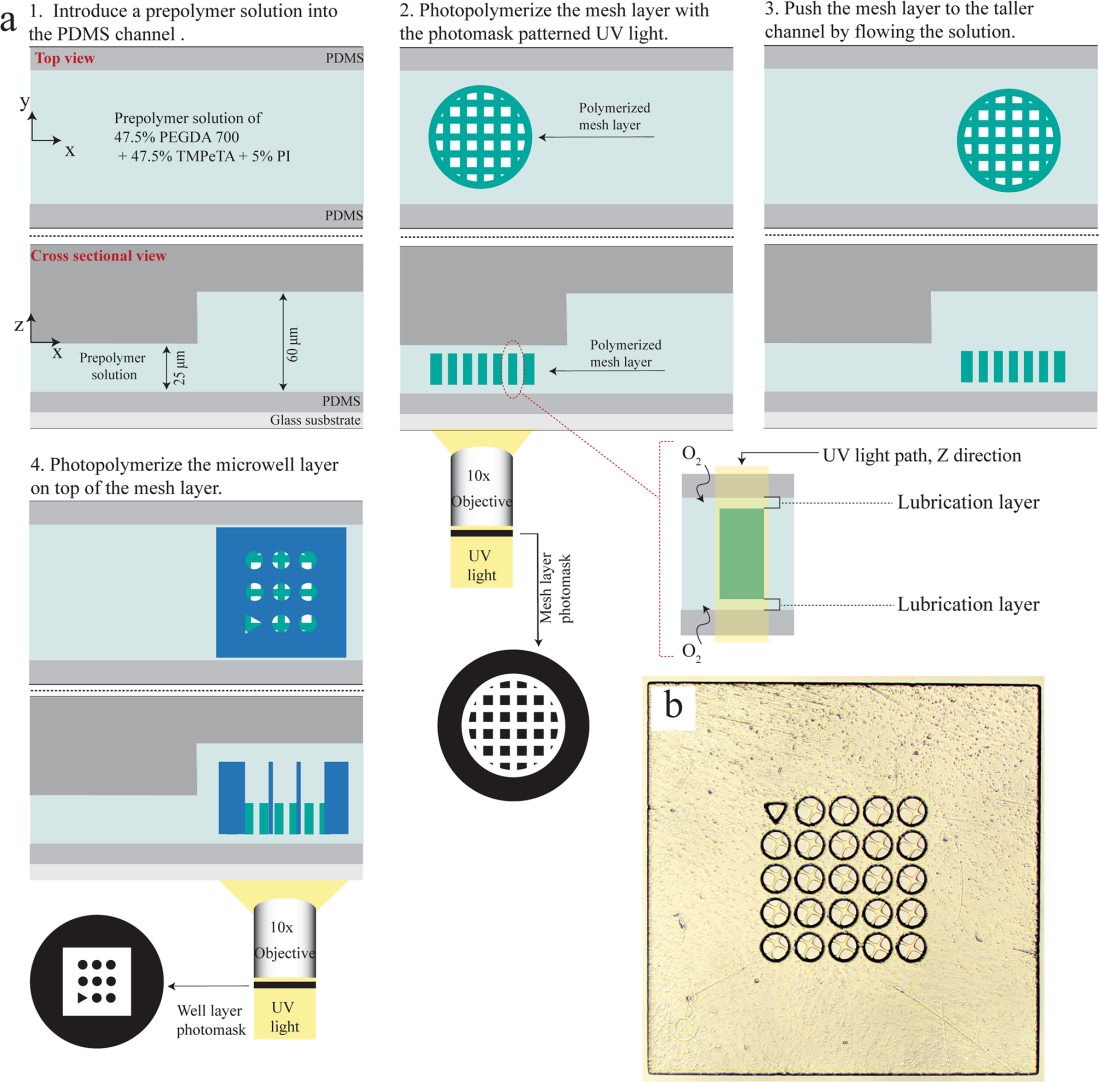
Fabrication schematic of sieved microwells with double layers using slit channel lithography. (**a**) A step channel is used to fabricate the two layers. The mesh layer is fabricated in the shorter region of the channel and moved to the taller region of the channel where the microwell layer is polymerized over it. (**b**) Bright field image of a resulting microwells, with a mesh pore size of 50 µm and a well diameter of 100 µm and overall microwell hydrogel length of 1.5 mm.

We fabricate sieved microwells by adapting slit channel lithography^36^ in a PDMS step channel with two different heights (Figure S1a). A thinner mesh layer is made in the shorter channel and pushed to a well-fabrication site for a thicker microwell layer to be made in the taller channel. The area nearest to the inlet of the channel is 25 μm in height, while halfway along the channel the height increases to 60 μm. The PDMS step channel is filled with a prepolymer solution to begin, and the first layer, the mesh layer, is polymerized using UV light through a photomask and a 10 × objective in the 25 μm tall region of the channel (Fig. 1a). The photomask and objective are used to define the shape and size of the mesh, while the channel height determines the height of the mesh layer resulting in a height that is approximately ~ 5 μm less than the channel height with the applied UV dose. This gap arises due to the oxygen inhibition layer at the top and bottom of the PDMS channel which prevents polymerization, in addition to acting as a lubrication layer to facilitate the movement of the mesh following polymerization. Additionally, the UV exposure time is used to regulate the degree of polymerization, controlling polymerization in both the vertical and lateral directions. The second step (Fig. 1a) involves moving the polymerized mesh from the 25 μm-tall area to the 60 μm-tall area of the channel for the well-layer fabrication. The mesh layer is positioned on the fabrication site and is aligned in the center of the microwells to be created. The photomask is changed to correspond to the microwell design and the microwell layer is polymerized overtop of the mesh using the same conditions as the previous step. To allow for a larger margin of error when centering the two layers, we design the body size of the mesh layer to be smaller than the microwell layer in addition for the body shape to be circular so that arbitrary rotation in the orientation of the mesh layer is irrelevant.

The final microwell array is 1.5 mm in length and ~ 55 μm in height and contains an array of large pores that are lined with smaller pores along the bottom of each, creating a series of sieve-like microwells (Fig. 1b). The average height of the mesh and microwell layers were 20 μm and 55 μm, respectively, resulting in an approximate well depth of 35 μm. The controlled height provided by the step channel allows for the reproduction of this depth throughout. Thus, we can simply tune the depth of wells by the height difference of the step channel. This method allows one to make a range of custom microwells by simply varying the photomasks, exposure time or objective in order to capture an assortment of particle sizes.

**Figure 2:**
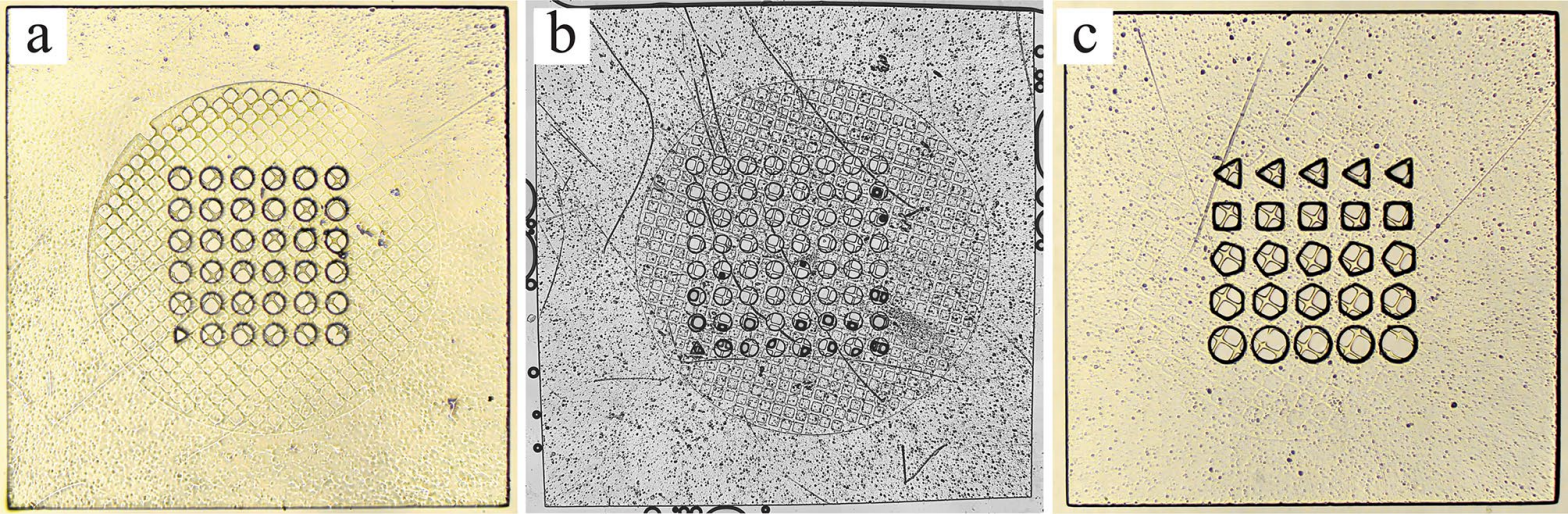
Bright field images of sieved microwells with various quantity, shapes and sizes of wells. (**a**) Microwells with a well diameter of 60 µm and pore length of 30 µm with an overall microwell array length of 1.5 mm. (**b**) Microwells made using 5 × objective to fabricate a larger microwell array with 63 pores and an overall length of 3 mm. The features sizes of the wells are 100 µm in diameter and 50 µm pore length. (**c**) Various well shapes can be made using a different photomask to create triangular, square, pentagonal, hexagonal and circular pores with a characteristic diameter of 100 µm and a pore length of 40 µm.

The microwell pores can be made into numerous shapes and sizes for a variety of purposes. By changing the photomask design the well diameter can be tuned from 30 to 100 μm (Fig. 2a), while also being made into various shapes, including triangular, square, pentagonal and hexagonal (Fig. 2c). Furthermore, the objective can be leveraged to create larger microwell arrays. In our current setup, when using the 10 × objective, the maximum length of uniform polymerization is 1.5 mm in length, which limits the overall length of the microwell array at this objective to 1.5 mm. By lowering the objective to 5 × , the focusable area increases, producing larger microwells with arrays up to 3 mm in length. Therefore, by using the same photomasks as the ones used at 10 × , the dimension of the microwells will be doubled at 5 × . For example, a pair of photomasks used to create a well diameter of 50 μm, mesh pore length of 25 μm and overall length of 1.5 mm at 10 × would fabricate a microwell with a well diameter of 100 μm, 50 μm mesh pore length and an overall length of 3 mm at 5 × (Fig. 2b).

**Figure 3:**
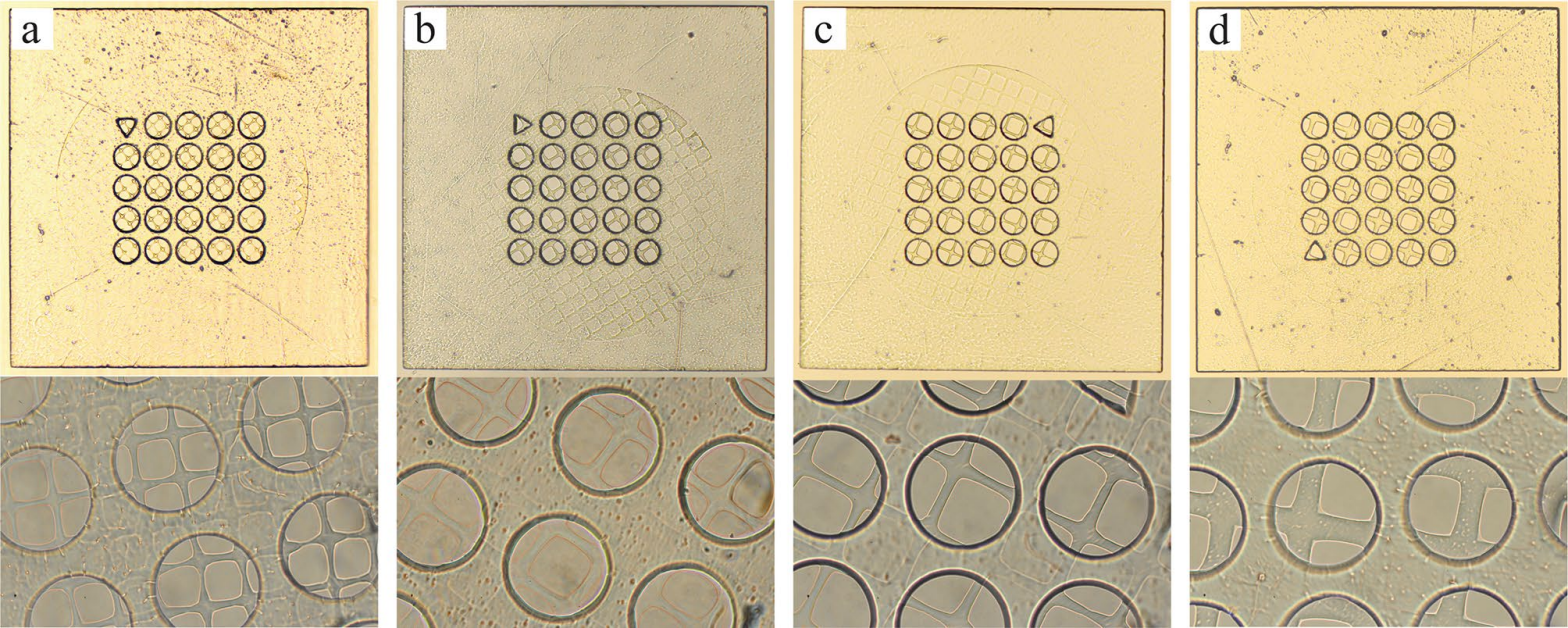
Bright field images of sieved microwells with 100 μm wells and mesh sizes controlled using different feature-sized photomasks, resulting in pore widths of approximately: (**a**) 40 μm, (**b**) 50 μm, (**c**) 60 μm, and (**d**) 60 μm with a 20 μm spacing.

**Figure 4:**
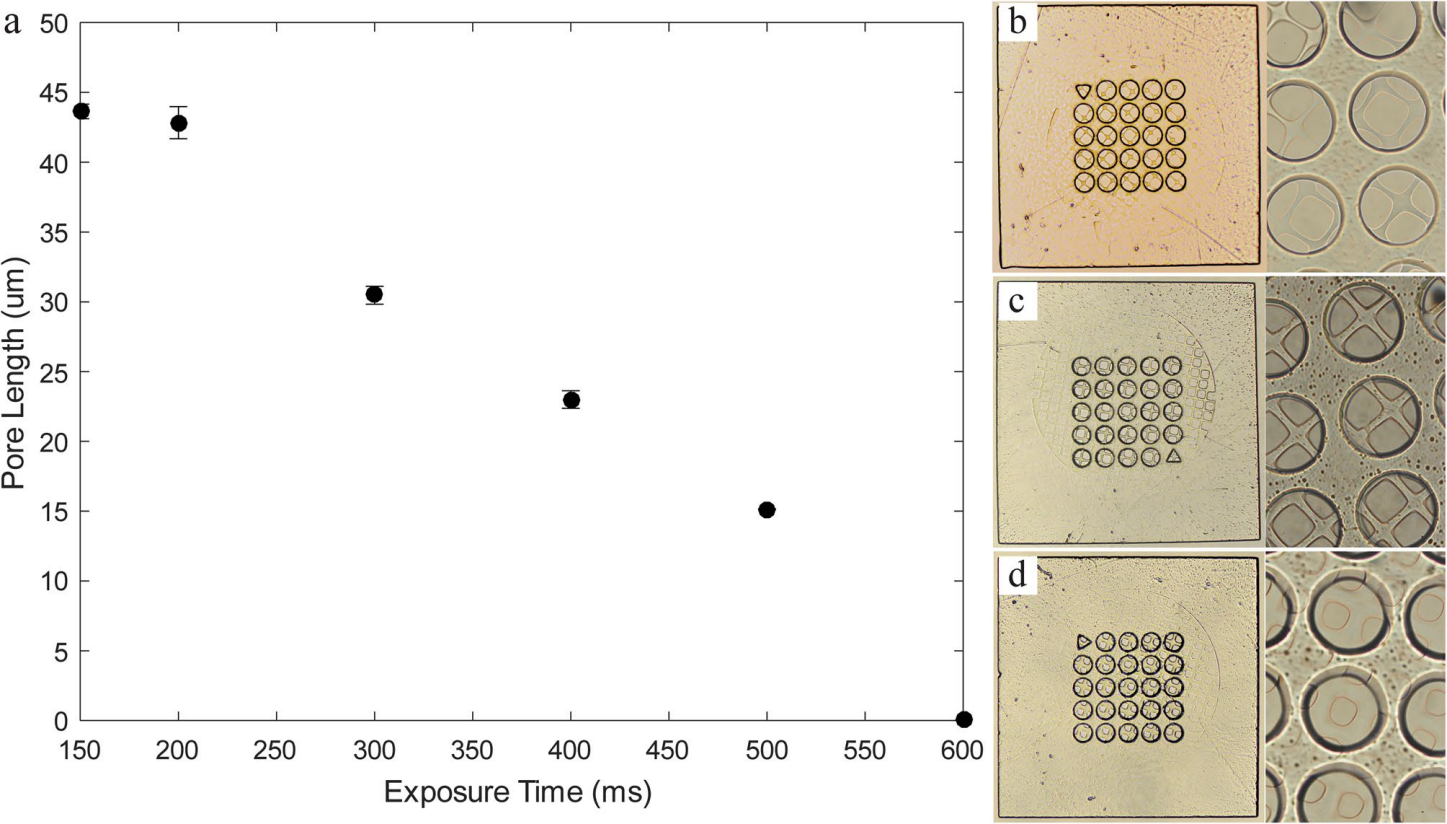
Mesh pore control with UV dose: (**a**) plot of the size of mesh pore reduction with an increase exposure time, resulting in completely closed pores at 600 ms. Bright field images of microwells with 100 µm wells and mesh sizes controlled by changing the exposure time, using times of: (**b**) 150 ms, (**c**) 250 ms, and (**d**) 500 ms.

Following the selection of an appropriate well shape and size, we control the mesh pore size using two different techniques. The first method uses various feature sizes of photomask to create a range of pore lengths from 20 to 60 μm (Fig. 3a–c) with a fixed UV dose, in addition, to being used to define the spacing between the pores (Fig. 3d). However, this approach requires an extensive number of photomasks for each size and spacing. The resulting pore size can slightly differ from the photomask because the focal plane of the objective is manually adjusted. The second method involves controlling the mesh pore size based on the exposure dose (Fig. 4a–d). The UV exposure dose is used to control the polymerization and therefore can be manipulated to vary the mesh pore size using a single photomask. As the UV exposure time at a fixed UV intensity is increased, polymerization occurs outside the UV projected zone because of free radical diffusion which results in a smaller mesh pore (Fig. 4a). Using UV dose control with a single photomask, we can create a range of mesh pores from completely closed to 43 μm pores in length. When increasing the exposure time over 200 ms, the pore length significantly decreases until it closes at 600 ms (Fig. 4a). However, any exposure time less than 150 ms results in low polymerization, leading to the shape failure where some parts of the mesh spacer are broken (Fig. 4b).

**Figure 5:**
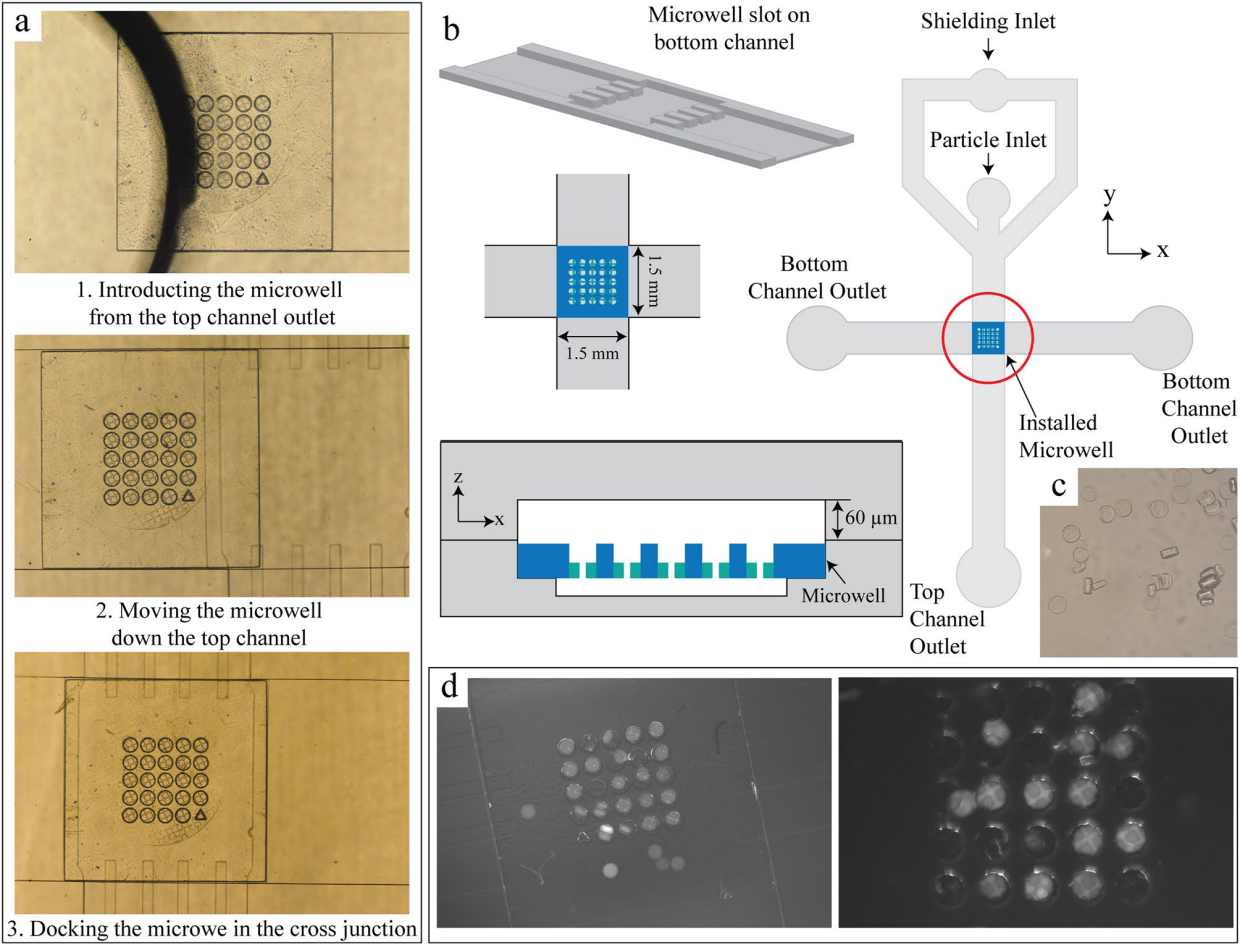
Installation and capture of particles in a microfluidic double channel with a 1500 µm width. (**a**) Installation of the microwells through the outlet of the top channel with a width is 1500 µm and a height of 60 µm. The microwell array is flowed through the channel to the intersection of the top and bottom channel where is fits into the microwell site. (**b**) Schematic of the cross section of an installed microwell and top view of the bottom channel. (**c**) Hydrogel disk particles with diameter of 80 μm and height of 28 μm. (**d**) Representative fluorescent images of particles captured in the microwell using a flow rate of 70 μL/min (left) and 50 μL/min (right).

To install the resulting sieved microwell into a microfluidic channel, we first exploit the bondless technique developed in our group^37^ without an applied magnetic field (Figure S1b). We flow a selected microwell array into a pre-assembled PDMS channel with a cross-flow configuration (Fig. 5a-b). The bottom channel is designed to have a seating area for the microwells. For the cross-flow channel assembly prior to the microwell installation, the top channel is placed perpendicularly over the seating area of the bottom channel and bonded together using partial curing. After the completion of the PDMS channel, we introduce the sieved microwells into the sitting site of the cross-junction of the top and bottom channels. During this step we ensure the microwell is facing upwards by orienting the triangle on the microwells in the correct direction (Fig. 5a). The top channel is designed to be 60 μm high and 1500 μm wide to accommodate the height and width of the microwell. Microwells are fabricated to be smaller than the sitting site since they expand to approximately 3% of their original size in a buffer solution of 5% Tween 20 which is used for particle trapping. We take the expansion into consideration when synthesizing the microwells to ensure that they fit right into the seating site. The microwells settle into the slot and is supported with a series posts along the bottom channel. The microwells thickness must be shorter than the height of the sitting site to prevent microwells from shearing off. Once the microwell array is docked, hydrodynamic pressure secures it down during flow operation.

For the microwell capture ability with this cross-flow configuration, we flow cylindrical particles with a density of approximately 10^4^ particles/mL. We fabricate the particles using stop flow lithography^38^. The microwell pores are 100 μm in diameter with a 40 μm mesh length and therefore the particles are made to be 80 μm in diameter and 28 μm in height to allow for single particle trapping (Fig. 5c). We flow the particles along the top channel using flow rates of 50–100 μL/min and capture them in the microwells. Untrapped particles continue to flow down to the outlet of the top channel. In the outlet of the bottom channel, there are no particles collected since particles are sieved along the flow path to the bottom channel. A shielding flow along the sides of the top channel is used at the same flow rate of the particles to guide the flow directly over the wells. With this range of flow rate, we obtain up to 49 ± 9% occupancy (Fig. 5d). The success of this device is measured by the well occupancy which is measured by the number of single particles capture in a well compared to the total number of wells. This poor performance is a result of the combination of the taller top channel used with the low flow rates applied. In these conditions, many particles pass over the microwells and tend to accumulate on top of the microwells. Although the freestanding microwell displays a low trapping ability in our current operation conditions, one beneficial feature of this configuration is that the microwell with captured targets can be retrieved out from the channel for post-processing.

Applying higher flow rates with shorter top channels can improve the trapping performance; however, the top channel height and flow rate are restricted. The height of the top channel should be greater than that of the microwell array for it to be installed through the top channel. At higher flow rates, the microwell array is prone to being sheared out since the array is not chemically nor physically attached. This microwell array dislocation can be severe when the array fitting is slightly off creating a small gap at the cross-junction of the microwells between the top and bottom channel. In our current manual system, we are able to operate flow rates of 50–100 μL/min without the microwell array fitting failure. Automatic fabrication and installation can improve microwell array fitting and allow for higher flow rates.

**Figure 6:**
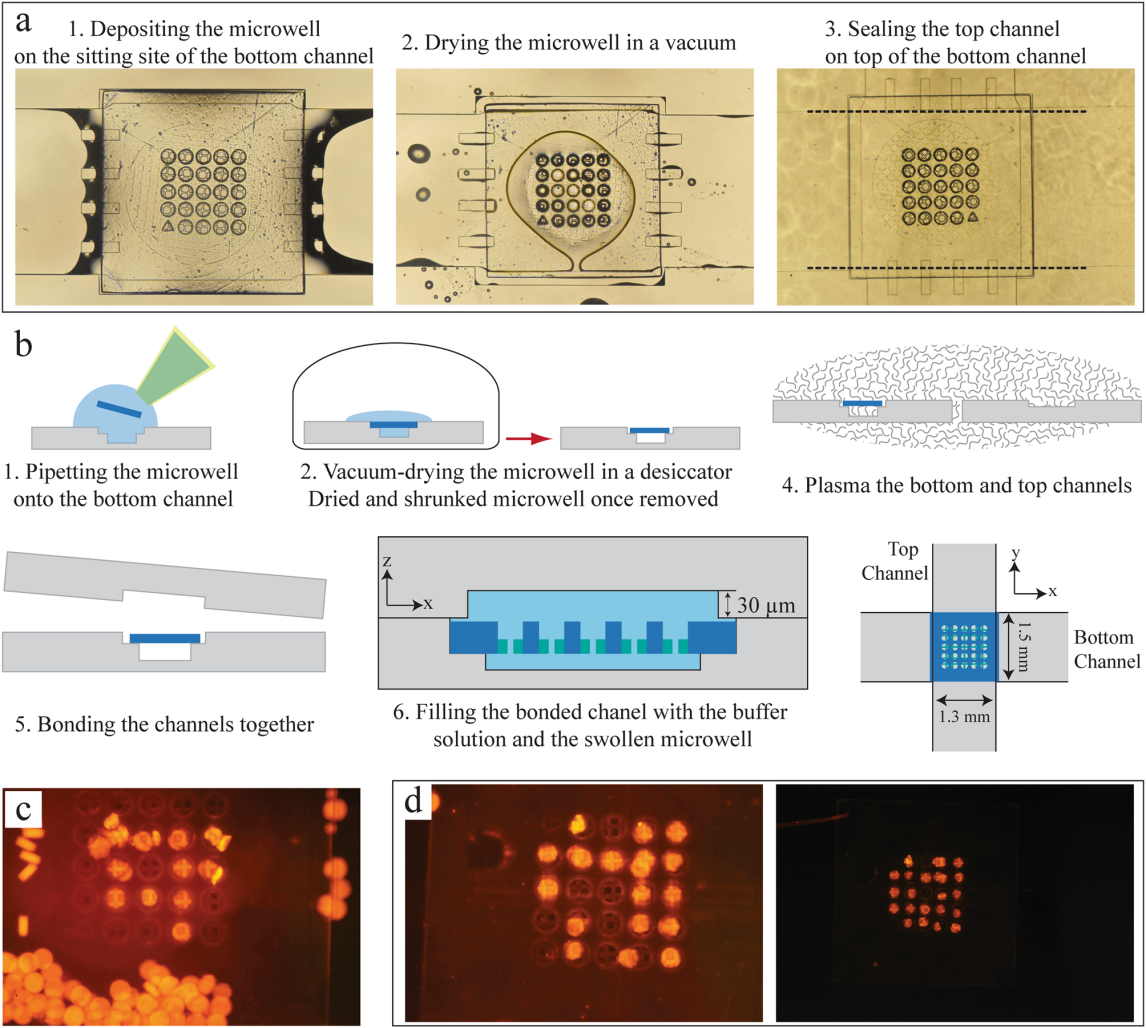
Installation and capture of particles in double channel with 1300 µm wide top channel. (**a**) Bright field images of three sequences and (**b**) schematic for the microwell installation: the microwell array is loaded onto the open bottom channel and liquid is evaporated using partial vacuum. The top channel, with a 1300 µm length and 30 µm height, and bottom channels are plasma treated (20 W for 30 s) and aligned to sandwich the microwell in place. (**c**) Representative fluorescent images of the particles captured using a flow rate of 50 μL/min, with numerous particles scatter along the surface. (**d**) Representative fluorescent images of the particles captured using a flow rate of ~ 9 mL/min for seven one-second intervals.

To reduce the top channel height and better secure microwells at higher flow rates, we develop a new assembly route (Fig. 6a,b) for microwells installation. In this route, a lower top channel height of 30 μm is used to bring particles in closer contact with the microwells, which prevents the microwells from being installed through the top channel. Using the same bottom channel as previously described, a microwell array is pipetted onto the seating area of the bottom channel and oriented so the wells are upwards. The bottom channel, with the microwells, is placed in a desiccator to remove any excess liquid. Both the top and bottom channels are then plasma treated and bonded together. The top channel was redesigned to be 1300 μm wide, which is slightly narrower than the width of the bottom channel. In this configuration, the microwell is physically constrained by the top channel since overlapping the top channel over the microwell prevents the microwell from sliding out of the seating area, allowing it to withstand higher flow rates. After assembling the channel, a 5% Tween-20 solution is introduced into the channel ensuring the microwells swell back to its’ original width of 1500 μm (Fig. 6a).

First, we applied the same flow range of 50–100 μL/min in the narrower top channel resulting in an average well occupancy of 46 ± 18%, with the highest results reaching up to 73%. Nevertheless, a large number of particles accumulate along the edges of the microwell resulting in a congestion of particles over the surface of the microwell array, (Fig. 6c) which was also observed in the previous configuration of the freestanding microwells. Using the maximum flow rate of the syringe pump (purging mode), approximately 9 mL/min, for one second intervals resulted in an increased well occupancy after each repetition. After seven one-second intervals at the maximum flow, the well occupancy of the microwells was 88 ± 6% (Fig. 6d) with a low concentration of disk particles introduced to the microwells. To increase the well occupancy, a high concentration of particles can be used or untrapped particles from the outlet can be flowed back to the microwells. We also observed no particle accumulation on top of the microwells.

Although this demonstration is shown with a small number of wells, 24 wells, a higher number of wells can be achieved and operated by installing multiple microwells in a parallel manner. This multi-parallel configuration has been demonstrated in our previous work^37^. This parallel configuration is commonly used in a membrane separation to increase separation efficiency. In addition, the total number of wells can be increased by enlarging the dimension of the microwell array by utilizing a lower magnification objective (Fig. 2b) or contact lithography. When a small pore size of wells is used, without increasing the microwell dimension, a minimum 20 × 20 array of wells can be introduced in a 3 mm × 3 mm microwell (Fig. 2b).

## Conclusions

We have designed a sieved microwell array fabricated in a simple two-step process using slit channel lithography which enables rapid fabrication of microwells. This fabrication method allows for a wide range of well and mesh sizes and shapes to be easily made by changing the photomask, objective or UV exposure time at constant power. Installing the microwells in a cross-flow microfluidic device significantly improves the well occupancy into the wells using hydrodynamic force through the open pores at the bottom of the wells. Furthermore, the installation methods of the microwells into the microfluidic device demonstrates the versatility of this arrangement. One method permits the easy retrieval of the microwell from the channel, which can facilitate isolation of specific cells or particles for further study, while the other allows for higher flow rates to be used which significantly reduces the capture time to under 10 s and occupancy of the wells to 87%. The device allows for numerous applications beyond particle capture, like enhancing single cell analysis by providing uniform nutrient dispersion throughout the wells, as opposed to relying of diffusion in conventional wells, in addition to allowing for seamless aqueous media changes in this device. To optimize and better utilize our porous-microwell trapping-system, comprehensive parameter studies such as flow rate, and well size and shape on the capture efficiency should be followed.

## Experimental section

### Slit channel lithography

Slit channel lithography^35^ was used to fabricate the microwells. An inverted microscope (Axio Observer, Zeiss, Germany) was equipped with a 10 × /0.3 objective (N-Achroplan, EC plan-Neofluar and korr LD Plan-Neofluar, Zeiss) and connected to either, a 365 nm LED (Super High-Power LED Collimator, Mightex, USA) as a near UV source, or a 405 nm LED (Super High-Power LED Collimator, Mightex, USA) as a violet light source, and a UV shutter (Lambda SC, Sutter Instruments, USA) for controlling the exposure time during the polymerization process. The supplied current to the LEDs and output was set to 10.4 A and 80% power for the near UV light and 13 A and 100% for the violet light. To control the flow rate of prepolymer solution in the PDMS channel, a pneumatic solution feeding system was serially connected to a pressure regulator (type 100LR, ControlAir, USA), a three-way solenoid valve (model 6014, Burkert, Germany) and the PDMS channel. Both the UV shutter and solenoid valve were controlled using Labview (National Instruments, USA) through a digital controller (NI-9472, National Instruments, USA) to computerize the control of prepolymer flow and near UV exposure time. Transparency photomasks were designed using AutoCAD and printed at a resolution of 25 000 dpi (CAD/Art Services, OR, USA).

### Microwell and hydrogel particle synthesis

The microwells are synthesized from a prepolymer solution of 47.5% poly(ethylene glycol) diacrylate (PEG-DA 700, Sigma-Aldrich, Germany), 47.5% trimethylolpropane ethoxylate triacrylate (TMPeTA 428, Sigma-Aldrich) and 5% 2-hydroxy-2-methylpropiophenin photoinitiator (Darocur 1173, Sigma-Aldrich). To make microwells with a 1.5 mm length, a PDMS step channel, with a width of 7 mm and a height of 25 and 60 μm, was filled with prepolymer solution. Once filled, the prepolymer solution flow is paused and the UV light (365 nm) is illuminated through the mesh photomask and 10 × objective for 200 ms, in the 25 μm area of the channel. The prepolymer solution flow is resumed for 50 ms to move the mesh layer to the 60 μm area of the channel. The mesh layer is centered in the focal area, and the UV light is exposed through a microwell photomask and 10 × objective for 200 ms. The prepolymer solution flow is resumed to flush the microwell into the outlet. The microwells is washed three times using denatured alcohol (Histoprep 95%, Fisher Scientific), and three times using deionized water with 5% ﻿polyethylene glycol sorbitan monolaurate (Tween 20, Sigma-Aldrich) and stored in this final aqueous solution. The 3 mm microwells are synthesized using the same procedure as previously, however, a prepolymer solution of 49.5% PEG-DA 700, 49.5% TMPeTA 428 and ~ 1% phenyl(2,4,6-trimethylbenzoyl)phosphine oxide photoinitiator (Irgacure 819, Sigma-Aldrich) is polymerized using a 5 × objective and violet light (405 nm) for 25 ms. ﻿Hydrogel particles were produced in a PDMS channel 2 mm in width and 30 μm in height using 95% poly(ethylene glycol) diacrylate (PEG-DA 700, Sigma-Aldrich) and 5% 2-hydroxy-2-methylpropiophenon photoinitiator (Darocur 1173, Sigma-Aldrich) with a 20 × objective and 30 ms exposure of UV (365 nm). Fluorescent particles were made by adding 5% Rhodamine B acrylate (1%, Sigma-Aldrich) to the particle prepolymer solution prior to polymerization. To transfer the microwells to the microfluidic device, a pipet with cut 200 μL pipette tip (VWR, USA) was used.

### Microfluidic step channel fabrication

The microfluidic step channels were fabricated using soft lithography^38^. The design of the channels was drawn using computer-aided design software (AutoCAD, Autodesk Inc.) and printed ﻿with a micro-pattern generator (µPG 501, Heidelberg Instruments, Germany) on a glass substrate. SU-8 2025 photoresist (Microchem, USA) was spin-coated onto a 4-in. diameter silicon wafer (University Wafer Inc., USA) and the silicon wafer was exposed to UV light through a photomask onto the patterned glass substrate; then, unexposed photoresist was removed with the developer solution from the silicon wafer to construct the positive master mold. The bottom-channel master mold was fabricated by repeating SU-8 spin-coating and UV light exposure twice with the second photomask in a mask alignment system (EVG620, EVG, USA) for the double layer construction. Poly(dimethylsiloxane) (PDMS, Sylgard 184, Dow Corning, USA) precursor with a 10:1 ratio was poured onto the surface of the silicon master mold baked at 65 °C. The channels are bonded using two methods: (1) To partially cure the PDMS, the mold was removed after 30 min. The partially cured PDMS was peeled off and aligned using a stereo microscope (Stereo Microscope Z10, USA). The assembled microchannel was baked at 65° for at least 1 h to completely cure the two layers; (2) To bond the layers using plasma treatment, the channels were fully cured at 65 °C overnight. The channels are placed in a plasma chamber (Tergeo-plus, PIE Scientific, USA) at 20 Watts for 30 s. The channels are aligned using the same stereo microscope and baked for an additional hour to ensure full bonding.

### Imaging

Bright-field and fluorescence images of the microwells and PDMS devices were taken using an inverted microscope (Axio Observer, Zeiss, Germany) with 5 × objective lenses, and a digital SLR camera (D300s, Nikon). For fluorescence imaging the green filter (XF100-2 Omegafilters, USA) was used. The 3 mm microwell was imaged using Axiocam 506 mono (Zeiss) on an inverted microscope (Axio Observer, Zeiss). All images were processed with Zeiss ZEN lite (Zeiss).

### Well occupancy determination

Using the fluorescent images of the captured particles, the number of wells filled with single particles are counted. This number is divided into the total number of wells (24) to determine the well occupancy. This is repeated with three different experiments to determine the average well occupancy for each arrangement.

## Supplementary information

Supplementary file1.
