# Supplementary material for: Rapid fabrication of sieved microwells and cross-flow microparticle trapping
Source: Sci Rep. 2020 Sep 24;10:15687. doi: 10.1038/s41598-020-72700-5 (PMC7518267; doi:10.1038/s41598-020-72700-5)
Supplement: Supplementary file 1 — Supplementary file1. [file 41598_2020_72700_MOESM1_ESM.pdf]

Supplementary Information

Rapid Fabrication of Sieved Microwells and Cross-Flow Microparticle Trapping

Lauren Romita[1,2,3], Shyan Thompson[1,2,3] and Dae Kun Hwang[1,2,3,*]

[1]Department of Chemical Engineering, Ryerson University
350 Victoria Street, Toronto, ON M5B 2K3, Canada
Email: dkhwang@ryeson.ca

[2]Keenan Research Centre for Biomedical Science, St. Michael's Hospital
30 Bond Street, Toronto, ON M5B 1W8, Canada

[3]Institute for Biomedical Engineering, Science and Technology (iBEST)
A Partnership Between Ryerson University and St. Michael's Hospital
30 Bond Street, Toronto, ON M5B 1W8, Canada

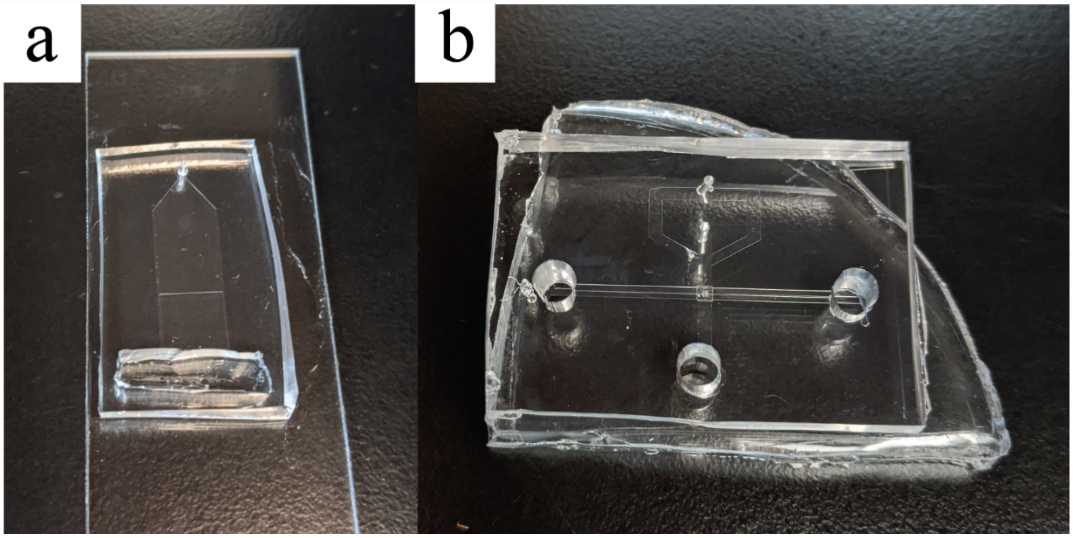

Figure S1. (a) Microfluidic step channel. (b) Cross-flow channel with microwell installed.
